# Acids Contained in Tobacco

**Published:** 1847-06

**Authors:** 


*Acids contained in Tobacco.*—M. Goupil's chemical investigations on this plant have confirmed Vauquelin's assertion of the presence of acid malate of lime in it; but he has gone farther in proving it to be in such quantity as to render tobacco an advantageous source from whence to obtain malic acid for the laboratory. From two and a-half pounds of the dried plant, about two or three ounces of acid malate of ammonia may be obtained. Except citric acid, M. Goupil has not found, in the numerous experiments he has made, any other organic acid. Last year, M. Barrell found, however, a peculiar bibasic acid in tobacco, which he denominated the nicotic acid, composed of C 3, H 4, O 3.—*Ibid, from An. de Chimie.*

